# Interleaved Learning in Elementary School Mathematics: Effects on the Flexible and Adaptive Use of Subtraction Strategies

**DOI:** 10.3389/fpsyg.2019.00086

**Published:** 2019-02-14

**Authors:** Lea Nemeth, Katharina Werker, Julia Arend, Sebastian Vogel, Frank Lipowsky

**Affiliations:** Department of Empirical Educational Research, Institute of Educational Science, Faculty of Human Sciences, University of Kassel, Kassel, Germany

**Keywords:** interleaved practice, comparison, subtraction strategies, mathematics, elementary school, strategy-specific adaptivity, flexibility

## Abstract

Empirical findings show that students are often not capable of using number-based strategies and the standard written algorithm flexibly and adaptively to solve multi-digit subtraction problems. Previous studies have pointed out that students predominantly use the standard written algorithm after its introduction, regardless of task characteristics. Interleaved practice seems to be a promising approach to foster the flexible and adaptive use of strategies. In comparison to the usual blocked approach, in which strategies are introduced and practiced successively, they are presented intermixed in interleaved learning. Thus, the students have to choose an appropriate strategy on the basis of every task itself, and this leads to drawing comparisons between the different strategies. Previous research has shown inconsistent results regarding the effectivity of interleaving mathematical tasks. However, according to the attentional bias framework, interleaved practice seems to be a promising approach for teaching subtraction strategies to enhance the students’ flexibility and adaptivity. In this study, 236 German third graders were randomly assigned to either an interleaved or blocked condition. In the interleaved condition the comparison processes were supported by prompting the students to compare the strategies (between-comparison), while the students of the blocked approach were encouraged to reflect the adaptivity of a specific strategy for specific subtraction tasks (within-comparison). Both groups were taught to use different number-based strategies (i.e., shortcut strategies and decomposition strategies) and the standard written algorithm for solving three-digit subtraction problems spanning a teaching unit of 14 lessons. The results show that the students of the interleaved condition used the shortcut strategies more frequently than those of the blocked condition, while the students of the interleaved condition applied the decomposition strategies as well as the standard written algorithm less frequently. Furthermore, the students of the interleaved condition had a higher competence in the adaptive use of the shortcut strategies and the standard written algorithm. A subsequent cluster analysis revealed four groups differing in their degree of adaptivity. Being part of clusters with a comparatively high level of adaptivity was positively related to the prior arithmetical achievement and, even more so, to the interleaved teaching approach.

## Introduction

There is a wide consensus among mathematics researchers and educators that the abilities to use various strategies for solving a problem (flexibility) as well as to use efficient strategies (adaptivity) are important mathematical competencies students should gain (National Council of Teachers of Mathematics [NCTM], 2000; Kilpatrick et al., 2001; Baroody and Dowker, 2003; Kultusministerkonferenz [Standing Conference of the Ministers of Education and Cultural Affairs of the Länder in the Federal Republic – KMK], 2004). However, several empirical findings indicate that elementary school students are often not capable of solving multi-digit subtraction problems flexibly and adaptively (Carpenter et al., 1997; Blöte et al., 2000; Selter, 2001; Torbeyns et al., 2006, 2009a; Heinze et al., 2009). Previous research has shown that students predominantly use the standard written algorithm after its introduction, regardless of any task characteristics, and then barely apply number-based strategies. Hence, the question for instructional approaches that foster students’ flexible and adaptive strategy use rises. Interleaved practice, in which the learning contents are intermixed, seems to be a promising approach to foster the flexible and adaptive use of subtraction strategies. In the following, we firstly operationalize the terms flexibility and adaptivity for our research. Then, we present different subtraction strategies that are well known in mathematics classrooms and review empirical results regarding the (adaptive) application of these strategies by elementary school students. Finally, the potential benefit of interleaved learning and the role of comparisons for the acquisition of subtraction strategies are deduced.

### Flexibility and Adaptivity

Reviewing the literature on the strategy use of elementary school students, a wide range of usage for the terms *flexibility* and *adaptivity* can be found. While some authors use the terms as synonyms (Baroody, 2003), others subsume both terms under flexibility (Thompson, 1999; Blöte et al., 2000). As Verschaffel et al. (2009) point out in their literature review, “it seems that the term ‘flexibility’ is primarily used to *switching (smoothly) between different strategies*, whereas ‘adaptivity’ puts more emphasis on *selecting the most appropriate strategy*” (p. 337). Accordingly, we use this definition to separate the two terms for our study. Hence, students need a repertoire of subtraction strategies to use them flexibly. Beyond that, flexibility itself is an “essential stepping-stone toward adaptivity” (Verschaffel et al., 2009, p. 339; see also Siegler, 1996).

To assess whether a specific strategy is adaptive for solving a specific subtraction task, a more precise definition is required. To decide, whether a strategy is adaptive, i.e., appropriate/efficient, for a certain subtraction task, we take a normative perspective following several other studies (e.g., Blöte et al., 2000, 2001; Heinze et al., 2009, 2018; Torbeyns et al., 2009a). Accordingly, we take (1) the number of solution steps, (2) the mental effort, and (3) the error rate into account when assessing the adaptivity of the used strategies. Therefore, whether a strategy is adaptive or not, does not depend on speed and accuracy. The accuracy of the strategy execution is measured by a separate variable since a student might use an adaptive strategy but make a calculation error. This separation of adaptivity and accuracy is useful to consider different aspects of solving subtraction problems. Threlfall (2002) as well as Verschaffel et al. (2009) criticize focusing solely on task characteristics to operationalize adaptivity since the strategy choice hinges on subject variables (e.g., the competence of a student to use a specific strategy) as well as the sociocultural context. However, we consider our normative perspective on adaptivity as appropriate for our research because the students were taught to use the mentioned normative criteria when deciding if a strategy is adaptive or not for a specific subtraction problem.

### Subtraction Strategies

There are several different classifications of subtraction strategies in the literature (for an overview, see Threlfall, 2002). For our research, we concentrated on a categorization of four idealized number-based strategies, which are widely known in the context of mathematics education, as well as the standard written algorithm as a digit-based strategy to solve multi-digit subtraction tasks (e.g., Wittmann and Müller, 1990; Threlfall, 2002; Benz, 2007; Heinze et al., 2009, 2018; Verschaffel et al., 2009; Padberg and Benz, 2011; Fierro, 2013; Bassarear and Moss, 2016; Kupferman, 2016; Schipper et al., 2017). The number-based strategies include two decomposition strategies (stepwise strategy and split strategy) and two shortcut strategies (compensation strategy and indirect addition, Table 1).

**Table 1:** Overview of the different subtraction strategies.

1 Number-based strategies	
1.1 Decomposition strategies	
1.1.1 Stepwise strategy	1.1.2 Split strategy
654 - 328 = 326	756 - 423 = 333
654 - 300 = 354	700 - 400 = 300
354 - 20 = 334	50 - 20 = 30
334 - 8 = 326	6 - 3 = 3
**1.2 Shortcut strategies**	
**1.2.1 Compensation strategy**	**1.2.2 Indirect addition**

547 - 399 = 148	452 - 449 = 3
547 - 400 = 147	449+ 3 = 452
147 + 1 = 148
**2 Digit-based strategy: standard written algorithm**	
725	
-453	
1	
----------	
272	

**FIGURE 1:**
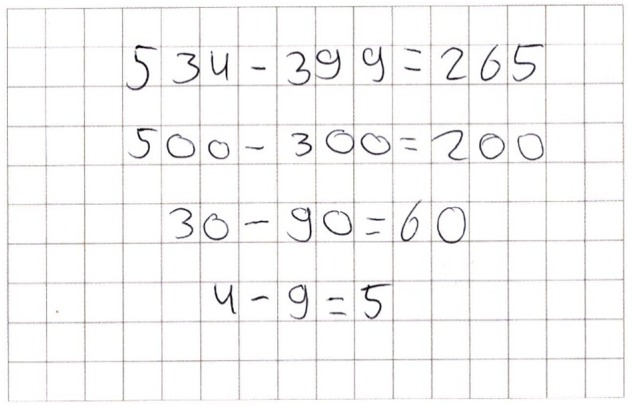
Typical mistake when using the split strategy.

Before the introduction of the standard written algorithm, students use the decomposition strategies most frequently to solve subtraction tasks, whereby the stepwise strategy is used most often (Blöte et al., 2000; Selter, 2001; Benz, 2007; Heinze et al., 2009). This may be due to the fact that the stepwise strategy can be used as a default procedure, i.e., as a strategy to solve all multi-digit subtraction tasks with, and that there are no obvious task characteristics marking that this strategy is efficient. Moreover, the stepwise strategy is often the only number-based strategy taught in traditional arithmetic classrooms before the standard written algorithm is introduced (Heinze et al., 2009). The second most used strategy is the split strategy. This strategy can cause difficulties solving subtraction tasks. Subtraction problems in which a digit of the subtrahend is greater than the corresponding digit in the minuend cause negative interim results which can lead to calculation errors. Meseth and Selter (2002) showed that 30% of the calculation errors of three-digit subtraction problems are due to the consequent subtraction of the smaller number from the greater number (Figure 1). Furthermore, it has been shown that even those students who have not been taught the split strategy use it (Meseth and Selter, 2002). Thus, the split strategy should be a subject of discussion in elementary school classrooms to foster a greater understanding for its difficulties among students (Wittmann and Müller, 1990; Meseth and Selter, 2002; Wittmann, 2003).

Unlike the stepwise and the split strategy, the shortcut strategies, compensation strategy and indirect addition, are used relatively rarely in mathematics classrooms (Hirsch, 2001; Selter, 2001; Benz, 2007; Heinze et al., 2009). Both types of shortcut strategies require a deep understanding of number relations and of the connection of the arithmetic operations to adapt the numbers and operations flexibly to task characteristics (Torbeyns et al., 2009a). The compensation strategy is especially adaptive for subtraction tasks in which the subtrahend is close to a full hundred, while the indirect addition is adaptive when solving subtraction tasks with a small difference between the minuend and the subtrahend. Regarding subtraction problems fulfilling these characteristics, the shortcut strategies only need little computation and cognitive effort. However, previous empirical studies have shown that students rarely use these strategies if they have not been taught systematically (Hirsch, 2001; Selter, 2001; Heirdsfield and Cooper, 2004; Benz, 2007; Torbeyns et al., 2009a; De Smedt et al., 2010). Furthermore, Heinze et al. (2009) showed in their study with German third graders that students barely use the mentioned number-based strategies adaptively to solve three-digit subtraction tasks.

Besides the mentioned number-based strategies, children learn to solve subtraction tasks with digit-based strategies, i.e., the standard written algorithm (see Table 1). Studies have converged to the conclusion that students predominantly use the standard written algorithm after its introduction, regardless of task characteristics, whereas the number-based strategies are then rarely applied (Selter, 2001; Clarke et al., 2006; Csíkos, 2016; Torbeyns and Verschaffel, 2016; Torbeyns et al., 2017; Caviola et al., 2018). Thus, the standard written algorithm is barely applied adaptively by elementary school students but replaces the stepwise strategy as the new default strategy.

Concerning this matter, previous research has detected several reasons why students do not use calculation strategies adaptively. On the one hand, a limited strategy repertoire can have a negative impact on choosing an efficient strategy (Torbeyns et al., 2009a). On the other, the conceptual knowledge about numbers turned out to be a significant positive predictor, since the students need an understanding of the number system and the arithmetic operations to apply them efficiently (Torbeyns et al., 2006, 2017; Torbeyns and Verschaffel, 2016).

Although the mentioned studies detected deficiencies in the flexible and adaptive use of subtraction strategies by elementary school students, they predominantly conceptualized flexibility and adaptivity by a variable-centered view as numerical variables. The only known study following a person-centered view was carried out by Torbeyns et al. (2017). They detected different subtraction strategy use profiles, i.e., flexibility profiles, and revealed that only a small proportion of students can be characterized as flexible strategy users. By following such a person-centered approach, qualitative differences in students’ flexible and adaptive strategy use can be explored. However, no studies are known following a person-centered view on the adaptive use of different subtraction strategies.

### Interleaved Practice and the Role of Comparisons

Summarizing the studies mentioned in the section above, children barely use subtraction strategies flexibly and adaptively to solve multi-digit subtraction problems. This may be explained by the usual *blocked practice*, which is the common approach for teaching subtraction strategies in elementary school classrooms (e.g., Selter, 2001; Department for Education and Skills [DfES], 2006; Common Core State Standard Initiative [CCSSI], 2010; Lemonidis, 2016). In the blocked practice, the strategies are introduced and practiced successively (firstly the number-based, afterward the standard written algorithm). Students learning subtraction strategies according to the blocked practice are not encouraged to reflect which strategy is adaptive for a specific subtraction task since they already know the strategy they have to use before they read a subtraction problem due to the consecutive structure (Rohrer et al., 2015). Hence, students do not learn to discriminate task characteristics and to choose an appropriate strategy on that basis. An alternative to the usual blocked approach is the *interleaved practice*. In an interleaved approach the introduction and practice of the different calculation strategies are systematically shuffled. In the short-term, i.e., during intervention, this approach hampers learning. In the long-term, however, studies showed an advantage of interleaved practice on learning outcomes after the intervention (Dunlosky et al., 2013). On the one hand, this benefit of interleaved practice can be explained by the *spacing* of the teaching content since problems of the same kind are distributed across different lessons and/or assignments (Rohrer et al., 2015). Several studies – mostly laboratory studies – have shown that spacing has a positive effect on the learning outcomes of students – also for mathematics (e.g., Grote, 1995; Cepeda et al., 2006; Rohrer and Taylor, 2006, 2007; Dunlosky et al., 2013). The spacing of the teaching content can lead to a distributed retrieval from the long-term memory (*retrieval hypothesis*), whereas students in a blocked approach probably only recall information out of the working memory to solve a task (Dunlosky et al., 2013). On the other hand, the advantage of interleaved practice can be explained by the *discriminative-/contrast hypothesis* (Kang and Pashler, 2012; Birnbaum et al., 2013). Goldstone (1996) states that “frequent alternation of categories has the advantage of highlighting features that serve to distinguish categories. Conversely, infrequent alternation of categories has the advantage of highlighting information that remains constant across the members within a category” (p. 615). Referring to the attentional bias framework, interleaving high-similarity categories directs the attention toward hard-to-find differences between the categories (Carvalho and Goldstone, 2015). Regarding this, blocked studying of subtraction strategies probably facilitates noticing similarities of tasks within one strategy, while students of an interleaved approach have to choose an appropriate strategy on the basis of every task itself. Hence, interleaving subtraction strategies as categories with a high level of similarity can encourage students to draw comparisons and to discover differences between the strategies (Richland et al., 2007; Rohrer and Taylor, 2007; Birnbaum et al., 2013; Dunlosky et al., 2013; Lipowsky et al., 2015). Concerning this matter, it can be assumed that interleaved practice fosters different dimensions of strategy knowledge, i.e., how to use the different strategies correctly (procedural knowledge), but also when and why which strategy (conditional knowledge) should be used.

Empirical findings regarding the effectivity of interleaved practice in mathematics are inconsistent, and this is emphasized by Brunmair and Richter’s (2019) meta-analysis. This meta-analysis showed a small positive effect of interleaving mathematical tasks on students’ procedural knowledge. However, the results of the studies included in this meta-analysis vary strongly. While some found a positive effect of interleaved practice (Rohrer and Taylor, 2007; Taylor and Rohrer, 2010; Sana et al., 2017), others showed no effect or even a negative impact (Rau et al., 2010; Higgins and Ross, 2011). Hence, it can be assumed that the effectivity of interleaved practice in mathematics depends on the concrete design (e.g., implementation, characteristics of learning materials, similarity of categories).

Laboratory studies investigating the effectivity of interleaving mathematical tasks are predominant, whereas only few studies have been conducted in real educational settings. Two of the few studies investigated in classroom settings were carried out by Rohrer et al. (2014, 2015). Both revealed a benefit of interleaved practice over blocked studying in the tests carried out 1 day and again 30 days after the intervention.

The inconsistent results regarding the effectivity of interleaved practice in mathematics lead to the assumption that the concrete implementation in the educational setting plays a major role. As the attentional bias framework (Carvalho and Goldstone, 2015) illustrates, interleaving supports identifying differences among low-discriminability categories, while blocked learning highlights similarities within one category. However, Durkin et al. (2017) summarize that students rarely discover similarities and differences between categories on their own. To support the students in discriminating, it seems to be a promising approach to combine interleaved practice with explicit prompts to compare. There are numerous studies indicating that encouraging students to draw comparisons between solutions, strategies, and procedures in mathematics can foster procedural knowledge (Rittle-Johnson and Star, 2007, 2009; Star and Rittle-Johnson, 2009; Ziegler and Stern, 2014, 2016; Ziegler et al., 2018), conceptual knowledge (Rittle-Johnson and Star, 2009; Star and Rittle-Johnson, 2009; Ziegler et al., 2018), the flexible use of strategies (Rittle-Johnson and Star, 2007, 2009; Star and Rittle-Johnson, 2009; Rittle-Johnson et al., 2012), and it can also lead to a decrease in misconceptions (Ziegler and Stern, 2014, 2016; Ziegler et al., 2018). Hence, it seems to be reasonable to combine interleaved practice with explicit prompts to compare in order to support the students’ discrimination processes.

The mentioned studies on interleaved practice indicate that it can have a positive impact on students’ learning outcomes in real educational settings, but there is still insufficient research on the subject: A first weakness of the available studies is that they were mostly conducted in laboratory and/or with university or middle school students leading to a limited transferability of the effects on elementary school mathematics. Secondly, previous studies have predominantly used the procedural knowledge as the dependent variable, whereas the effect of interleaving on the flexible and adaptive strategy choice as a major goal of mathematics education was unconsidered. Concerning this, it can be assumed that the effectivity of interleaving mathematical tasks, with studies showing inconsistent findings, is higher when the students’ discrimination processes are supported by explicit prompts to compare (Carvalho and Goldstone, 2015).

### Research Questions

The ability to use different subtraction strategies flexibly and adaptively is a major goal of teaching arithmetic in elementary school. Even though there is a stronger consideration of number-based strategies in classrooms nowadays, students barely use them efficiently to solve subtraction tasks, but prefer to rely on the standard written algorithm after its introduction. Interleaved practice combined with explicit prompts to compare for supporting the discrimination processes (Carvalho and Goldstone, 2015) seems to be a promising approach to foster a greater flexible and adaptive use of subtraction strategies compared to blocked learning including prompts to compare within one strategy (i.e., whether one specific strategy is adaptive or not for a specific task). However, the efficacy of interleaved practice in elementary school mathematics on students’ flexible and adaptive choice of subtraction strategies has not been investigated yet. Therefore, the present study examines whether interleaved learning including prompts to draw comparisons between the strategies has a positive impact on the acquisition of subtraction strategies regarding their flexible and adaptive use based on four research questions.

(1) Does interleaved practice have a positive impact on the flexible use of subtraction strategies?
(2) Does interleaved practice have a positive impact on the adaptive use of each subtraction strategy?

We supported the discrimination processes evoked by interleaved practice through explicit prompts to compare in order to direct the attention of the students to the differences between the strategies. The flexible and adaptive application of subtraction strategies is expected to benefit from the intervention. A substantial amendment of this research consists in examining the adaptive use for each strategy separately facilitating a differentiated insight into the effectivity of interleaved practice.

(3) Are there clusters of students differing in the adaptive use of the newly acquired subtraction strategies?

Another goal of this study is to identify students with different adaptivity profiles. In addition to the first two research questions following a variable-centered approach, the third research question is taking a person-centered view. By this person-centered view which takes variability between and within the students into account, adaptivity profiles can be generated. Thus, it can be shown whether student subgroups can be identified that differ in the adaptive application of the different subtraction strategies. An exploratory approach will be used to pursue this question since no hypotheses about possible adaptivity profiles can be formulated in advance.

(4) Do the teaching approach and the prior arithmetical achievement predict the adaptivity profile of students?

On the basis of the cluster analysis, the fourth research question explores if being taught subtraction strategies interleaved or blocked is related to the cluster membership. It is expected that the probability of being grouped in a cluster with a high level of strategy-specific adaptivity is higher when having been taught subtraction strategies interleaved. Moreover, previous research has shown that the knowledge about numbers, number relations, and the arithmetic operations are central prerequisites for using subtraction strategies efficiently (Torbeyns et al., 2006, 2017; Torbeyns and Verschaffel, 2016). For this reason, the teaching approach as well as the arithmetical prerequisites are taken into consideration.

## Materials and Methods

### Design and Participants

In a 2 (group: interleaved vs. blocked) × 4 (time: before intervention, 1 day later, 1 week later, 5 weeks later) experimental study, German elementary school students were taught in either an interleaved or blocked condition in solving three-digit subtraction problems with different strategies. A total sample of 236^1^ German third graders from 12 different classes attending four Hessian elementary schools participated in this study. The classes were split, and the students were randomly assigned to one of the conditions. In this way, one half of the class learned the subtraction strategies blocked and the other half interleaved. The students themselves did not know they were taught differently. A precondition to be part of the study was that the subtraction up to 1,000 had not previously been introduced in class. The addition up to 1,000 had to be introduced. During the intervention (until T2), no regular mathematics lessons were held.

**FIGURE 2:**
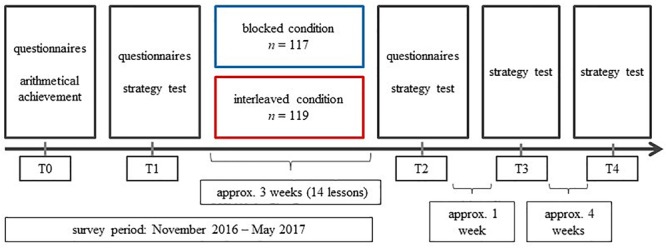
Design of the study.

The prior arithmetical achievement was measured at T0 in November 2016, i.e., before the intervention took place. The variables flexibility and strategy-specific adaptivity were measured immediately before the intervention (T1), immediately after the intervention (T2), and in two follow-up tests – 1 week (T3) and 5 weeks (T4) after the treatment (Figure 2).

The students involved in the study were aged from 8 to 10 years old (*M* = 9.06, *SD* = 0.41). About half of the participants (45.34%) were female. A total of 119 students were randomly assigned to the interleaved condition and 117 to the blocked one. Table 2 shows an overview of the prerequisites of the two groups. Different statistical tests were conducted, which did not reveal significant differences regarding the age of the students, *t*(231) = 0.80, *p* = 0.43, the proportion of female and male students, χ^2^(1) = 0.00, *p* = 0.99, and the prior arithmetical achievement, *t*(231) = 0.80, *p* = 0.87. As a MANOVA revealed, there were no significant differences between the students of the interleaved and the blocked condition before the intervention concerning how often they used the standard written algorithm, the split strategy, the stepwise strategy, and the indirect addition in the 11 tasks of the strategy test, *F*(5,230) = 0.38, *p* = 0.87, Wilk’s λ = 0.99, $\eta_{p}^{2}$ = 0.01. Another MANOVA showed no significant differences between the two groups regarding the strategy-specific adaptivity of the standard written algorithm, the stepwise strategy, the compensation strategy, and the indirect addition in the pretest, *F*(4,217) = 0.13, *p* = 0.97, Wilk’s λ = 1.00, $\eta_{p}^{2}$ = 0.00. The split strategy was not part of this analysis since it could not have been used adaptively in the strategy test (see the section “Flexibility and Strategy-Specific Adaptivity”).

**Table 2:** Prerequisites of the students separately for the interleaved and the blocked condition.

	**Interleaved**		**Blocked**	
Age *M* (*SD*)	9.04 (0.40)		9.10 (0.43)	
Female (%)	45.38%		45.30%	
Arithmetical achievement *M* (*SD*)	12.04 (5.65)		12.17 (6.02)	
Quantity of strategy use *M* (*SD*)	Written algorithm	0.68 (2.29)	Written algorithm	0.70 (2.47)
	Split strategy	0.66 (2.13)	Split strategy	1.05 (2.60)
	Stepwise strategy	6.40 (5.81)	Stepwise strategy	5.90 (4.86)
	Compensation strategy	0.68 (1.98)	Compensation strategy	0.61 (1.81)
	Indirect addition	0.12 (0.96)	Indirect addition	0.16 (1.00)
Strategy-specific adaptivity *M* (*SD*)	Written algorithm	3.08% (12.22%)	Written algorithm	4.08% (14.71%)
	Stepwise strategy	30.77% (24.73%)	Stepwise strategy	30.66% (25.69%)
	Compensation strategy	6.41% (20.22%)	Compensation strategy	7.15% (21.39%)
	Indirect addition	2.65% (16.15%)	Indirect addition	3.59% (17.42%)

### Treatment

The treatment included 14 lessons (à 45 min) and was conducted by four trained staff members who studied mathematics for elementary school. Each staff member taught the blocked as well as the interleaved condition in the same quantity. For an increased comparability of the lessons, a precise script was developed for each condition. This script contained detailed information on the time course of the lessons, the tasks, the expected behavior of the students, and possible teacher reactions, teacher questions, and possible action alternatives.

The main teaching goal of both conditions was to teach the students how to solve subtraction tasks adaptively. Therefore, the number-based subtraction strategies, including the decomposition strategies (split strategy and stepwise strategy) and the shortcut strategies (compensation strategy and indirect addition), and the standard written algorithm as a digit-based strategy were introduced and practiced in class. In addition to the introduction and use of the technical terms of the subtraction strategies, pictorial representations of animals^2^ were assigned to the different strategies as previous research has shown that labeling categories can support comparison mechanisms (Namy and Gentner, 2002). Moreover, the previously mentioned criteria in Section “Flexibility and Adaptivity” that were used to decide whether a strategy is adaptive or not (number of solution steps, mental effort, error rate) were taught to the students of both conditions to enhance their adaptive use of subtraction strategies. To support the students in arguing whether a specific strategy is adaptive for a given task, a poster containing these criteria was hung up in each lesson in the classroom.

In both conditions, the time spent on the strategies in classroom discussion and individual work was nearly equal. However, the time percentages differed between the strategies in both conditions: The time spent on the split strategy (about 55 min) was comparatively low in both conditions, since this strategy is error-prone (see the section “Subtraction Strategies”) and therefore, was only part of the teaching unit used to sensitize the students for potential difficulties. The time spent on the stepwise strategy, the compensation strategy, and the indirect addition was about 100 min each, and on the standard written algorithm with about 190 min even higher. While the time percentages for the strategies were equal in the two conditions, they differed in the order of the introduction and practice of the strategies. The first two lessons were equal for both conditions to activate relevant previous knowledge (knowledge of numbers: e.g., number relations on a number line, greater/less-comparisons) and to initiate a first approximation of using subtraction strategies in a clever way in a math conference, i.e., groups of students discussed which strategy is the most appropriate for solving a specific subtraction task. In the following lessons, the two conditions differed in the order of the introduction and practice of the strategies and the teaching activities (Table 3).

**Table 3:** Overview of the activities of each lesson.

	Activities	
Lesson	Blocked	Interleaved
1 & 2	• Activation of relevant previous knowledge about numbers (e.g., number relations on a number line, greater-/less-comparisons) • Introduction how to calculate cleverly • Math conference (students discuss in groups which strategy is the most clever one for a specific task)	

3	• Introduction and practice of the split strategy • Thematization of the difficulties the split strategy can cause • Within-comparisons (students have to decide whether the split strategy is adaptive for specific tasks or not)	• Introduction and practice of the split strategy • Introduction and practice of the stepwise strategy • Thematization of the difficulties the split strategy can cause • Between-comparisons (students have to decide whether the split strategy or the stepwise strategy is more adaptive for specific tasks)

4	• Introduction and practice of the stepwise strategy • Within-comparisons^3^	• Successive repetition and practice of the split strategy and the stepwise strategy • Between-comparisons

5	• Repetition and practice of the stepwise strategy	• Successive repetition and practice of the split strategy and the stepwise strategy • Between-comparisons • Introduction and practice of the compensation strategy

6	• Introduction and practice of the compensation strategy • Within-comparisons	• Successive repetition of the compensation strategy, stepwise strategy, and the split strategy • Between-comparisons

7	• Repetition and practice of the compensation strategy • Within-comparisons	• Introduction and practice of the indirect addition • Between-comparisons (stepwise strategy and indirect addition)

8	• Introduction and practice of the indirect addition • Within-comparisons	• Repetition of the indirect addition • Between-comparisons (stepwise strategy, compensation strategy, indirect addition)

9	• Repetition and practice of the indirect addition • Within-comparisons	• Introduction of the standard written algorithm

10	• Introduction of the standard written algorithm	• Repetition and practice of the standard written algorithm • Between-comparisons (compensation strategy and standard written algorithm)

11	• Repetition and practice of the standard written algorithm	• Successive repetition of the standard written algorithm and the compensation strategy • Between-comparisons

12	• Repetition and practice of the standard written algorithm • Thematization of typical mistakes when using the standard written algorithm	• Successive repetition of the standard written algorithm and the indirect addition • Between-comparisons • Thematization of typical mistakes when using the standard written algorithm

13	• Repetition and practice of the standard written algorithm • Within-comparisons	• Successive repetition and practice of the standard written algorithm and the split strategy • Between-comparisons (split strategy, compensation strategy, standard written algorithm)

14	• Successive repetition of the split strategy, the compensation strategy, the indirect addition, and the standard written algorithm • Within-comparisons for each strategy	• Successive repetition of the split strategy, the compensation strategy, the indirect addition, and the standard written algorithm • Between-comparisons

Due to the fundamental importance of the discrimination of contents for the interleaved practice (Carvalho and Goldstone, 2015), the strategies were not only taught and practiced in a mixed way. Furthermore, the students of the interleaved condition were explicitly prompted to compare the strategies, to reflect their adaptivity for specific tasks, and to explain why one specific strategy is more adaptive than the other (between-comparison). While the subtraction strategies were intermixed in the interleaved condition, they were taught successively in the blocked condition: first the number-based strategies, followed by the standard written algorithm. Another difference between the two conditions was that the students of the blocked condition were not prompted to draw comparisons between the strategies. However, the specific task characteristics that evoke each subtraction strategy were part of classroom discussions (within-comparison, i.e., students were prompted to decide whether a specific strategy is adaptive or not for a specific task) to support the advantage of blocked teaching highlighting similarities within one category.

Table 4 illustrates the differences between the two conditions in classroom discussions. Both examples are taken from the introduction of the indirect addition (frog strategy; interleaved: lesson 7, blocked: lesson 8) after the students had already practiced the application of this strategy.

**Table 4:** Examples for within-comparisons in the blocked approach and between-comparisons in the interleaved approach in classroom discussion.

	Blocked	Interleaved
Material	Subtraction tasks (413 – 409, 287 – 152, 579 – 348) solved solely with the indirect addition	Subtraction tasks (413 – 409, 287 – 152, 579 – 348) solved with the indirect addition and the stepwise strategy
Instruction	“You have solved many tasks using the frog-strategy. Now we want to find out, for which tasks it is clever to use the frog-strategy. Let’s have a look at the following tasks. When is it clever to use the frog-strategy?”	“You have solved many tasks using the frog-strategy. Now we want to compare the frog-strategy and the mouse-strategy. Let’s have a look at the first task. How did the frog solve the task? How did the mouse solve the task? Which strategy is more clever?”
Expected student behavior	The students recognize that the indirect addition is adaptive for tasks with a small difference between the minuend and the subtrahend. The students argue for or against the application of the indirect addition based on the discussed criteria (number of solution steps, error rate, cognitive effort).	The students recognize that the indirect addition is more adaptive than the stepwise strategy for tasks with a small difference between the minuend and the subtrahend. The students argue for or against the application of a specific strategy based on the discussed criteria (number of solution steps, error rate, cognitive effort).

**FIGURE 3:**
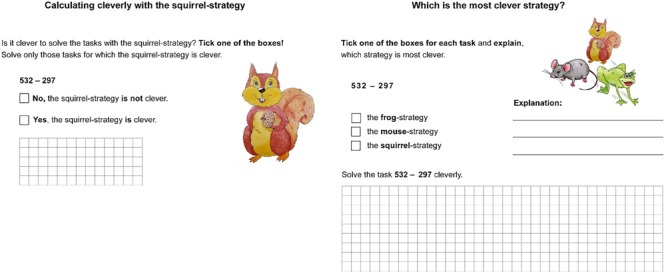
Examples for within-comparisons in the blocked approach (on the left) and between-comparisons in the interleaved approach (on the right) in individual work.

In each lesson, the students had to work on one to two worksheets that were developed for this teaching unit. The subtraction tasks of the work sheets were the same for both groups. Based on the worksheets, the students practiced either the application of the strategies procedurally or they were prompted to draw comparisons between (interleaved condition) or within (blocked condition) the strategies. Figure 3 illustrates the differences of the two teaching approaches during individual work. On the left is an example for the blocked condition (lesson 7). Here, the students have to decide whether a prescribed strategy (here: compensation strategy) is adaptive (clever) for solving different tasks or not. The example for the interleaved condition (lesson 8) on the right shows that the students have to decide which strategy is the most clever one for each task, and they need to explain why a specific strategy is clever (mouse as stepwise strategy, squirrel as compensation strategy, frog as indirect addition).

Furthermore, posters of the subtraction strategies including the animal illustrations and worked examples with complete solution procedures were hung up during the relevant lessons since they can support the students in discovering the characteristics and underlying rules of each subtraction strategy (Renkl, 2002). In addition, a mathematical lexical storage was provided for the students of both conditions to support them in reasoning. This lexical storage contained relevant mathematical terms and the corresponding explanations (e.g., minuend = the first number of a subtraction task, close together/small difference). The students got no homework in mathematics during the intervention and they were not allowed to take the materials home to avoid other influences on our treatment.

### Instruments

#### Arithmetical Achievement

**FIGURE 4:**
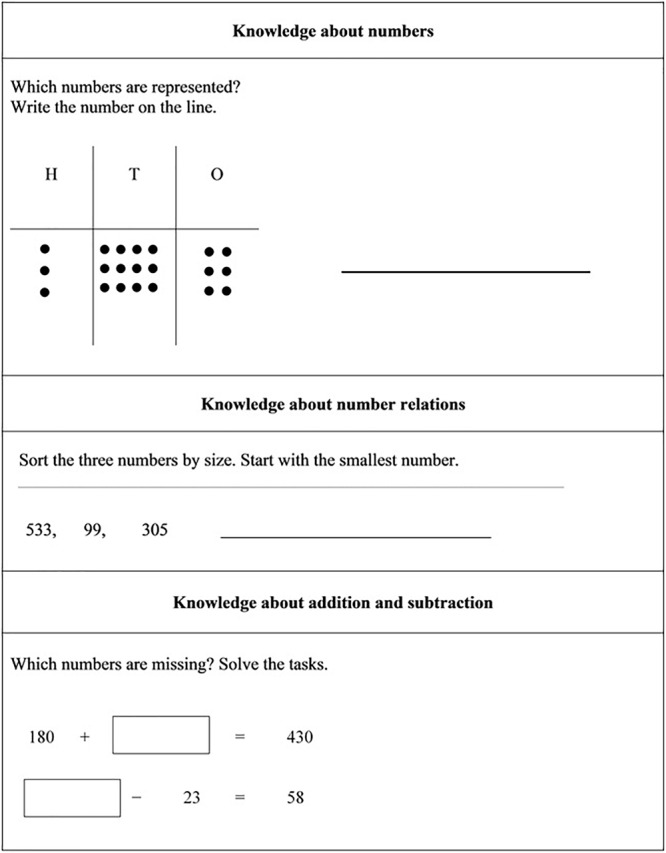
Sample tasks of the arithmetical achievement test. H, hundreds; T, tens; O, ones.

The arithmetical achievement of the students regarding their knowledge about numbers, number relations, about the relation of addition and subtraction, and competencies in calculating were measured at T0 (Figure 4).

The test consisted of 25 tasks and the students could have achieved a maximum of 25 points. To ensure that all students understood every task, the survey headers explained each task with a standardized test instruction. Students were required to solve the test in 36 min. On average, the students reached 12.10 points (*SD* = 5.82). The reliability of the test was satisfying (Cronbach’s α = 0.88).

#### Flexibility and Strategy-Specific Adaptivity

The dependent variables flexibility and adaptivity were measured at T1, T2, T3, and T4 using a subtraction strategy test. The test contained 11 items on each point of measurement assessing how (i.e., with which subtraction strategy) the students solve subtraction problems^4^. Six out of the 11 items were included in the test of each point of measurement, while the other five items varied to reduce potential memory effects. The varying items were developed parallel in respect of task characteristics and therefore, should represent the same competence (e.g., T1: 469 – 283, T2: 745 – 271, T3: 629 – 372, T4: 836 – 352; in all tasks, the tens-digit of the minuend is smaller than the tens-digit of the subtrahend). The prompt of the test was “Solve the tasks in a clever way. Write down how you solved the tasks.” The test took 28 min. The selected tasks evoked the mentioned number-based strategies (except of the split strategy) as well as the standard written algorithm. For most of the items using the indirect addition (four items at each point of measurement, e.g., 663 – 656) or the compensation strategy (four items at each point of measurement, e.g., 534 – 399) was most adaptive. The stepwise strategy and the standard written algorithm were considered to be almost equally adaptive for the three remaining items (e.g., 532 – 476). One exception here was the item which had a zero in the minuend (720–269) because zeros in the minuend often lead to calculation errors when using the standard written algorithm (Haylock and Cockburn, 2013). The stronger consideration of the indirect addition and the compensation strategy is based on empirical results showing that students rarely use those two shortcut strategies. Instead they focus on the stepwise strategy (before the introduction of the standard written algorithm; Blöte et al., 2000; Selter, 2001; Benz, 2007; Heinze et al., 2009) and on the standard written algorithm after its introduction (Selter, 2001; Clarke et al., 2006; Csíkos, 2016; Torbeyns and Verschaffel, 2016; Torbeyns et al., 2017), but rarely use them efficiently. The split strategy could not have been used adaptively since there was no task in the strategy test which could have been solved adaptively using this strategy. The main goal of dealing with the split strategy in this study was to enhance a greater understanding of the difficulties it can cause (see the section “Subtraction Strategies”).

To assess the students’ flexibility, their strategy use was coded by four trained coders independently guided by a standardized coding manual. This coding manual had been developed based on the coding manual of the TigeR-study (Heinze et al., 2018). The inter-coder agreement was very satisfying (κ ≥ 0.88). In cases in which the coders did not agree, a consensus was negotiated.

Besides coding the applied strategies, the adaptivity of all subtraction strategies was rated for each task in the tests. Two independent raters estimated the adaptivity dichotomously (0 = non-adaptive, 1 = adaptive). For the normative adaptivity rating, the following criteria were taken into consideration: number of solution steps, mental effort, and error rate. The inter-rater reliability was overall satisfactory (0.69 ≤ κ ≤ 1.00). If the raters did not agree, a consensus was negotiated.

In order to be able to assess the effectivity of interleaved practice on each subtraction strategy, the raw data of the adaptivity rating were restructured and the strategy-specific adaptivity was calculated. Since every strategy could not have been used adaptively in the same quantity, an index of the adaptive use of the different subtraction strategies at each point of measurement was generated by relativizing the sums of the actual adaptive use in consideration of (1) the potential adaptive and non-adaptive application at one point of measurement as well as (2) the actual, individual sums of the adaptive and non-adaptive use at one point of measurement.

This led to the following equation:

$$\begin{array}{c} Strategy-specific\;adaptivity=\frac{\frac{a_{a}}{a_{p}}}{\frac{na_{a}}{na_{p}}+\frac{a_{a}}{a_{p}}}\times 100\% \end{array}$$

with:

*strategy-specific adaptivity* relative proportion of the adaptive use of a specific strategy
*a_a_*                                             sum of the actual adaptive use of a specific strategy
*a_p_*                                             sum of the potential adaptive use of a specific strategy
*na_a_*                                           sum of the actual non-adaptive use of a specific strategy
*na_p_*                                           sum of the potential non-adaptive use of a specific strategy.

The procedure for calculating the strategy-specific adaptivity index is shown in the following example: The standard written algorithm could have been applied nine times non-adaptively and twice adaptively in the test 1 day after the intervention. If one student solved five subtraction tasks non-adaptively using the standard written algorithm and once adaptively, the relative proportion of the strategy-specific adaptivity would have been

$$\begin{array}{c} \frac{\frac{1}{2}}{\frac{5}{9}+\frac{1}{2}}\times 100\%\;=\;47.37\%. \end{array}$$

If students did not use a specific strategy at one point of measurement, even though it would have been adaptive, their strategy-specific adaptivity was set 0.00% for this specific strategy.

### Analysis

#### Research Questions 1 and 2

To address the first research question, whether interleaved practice has a positive impact on the flexible use of subtraction strategies, the frequency of use was summed up for every subtraction strategy at every point of measurement. The differences of the strategy distributions between the two conditions were determined by χ^2^-homogeneity tests for each point of measurement (T1, T2, T3, T4).

To address the second research question, whether interleaved practice has a positive effect on the adaptive use of the standard written algorithm, the stepwise strategy, the compensation strategy, and the indirect addition, 2 (group) × 4 (time) ANOVAs with repeated measures (T1, T2, T3, T4) were conducted for each strategy. When the assumption of sphericity was violated, the Greenhouse–Geisser correction was used. Pairwise comparisons between the points of measurement were calculated in cases of a significant time effect with Bonferroni adjustments for multiple comparisons to identify between which points of measurement the significant differences occurred. In cases of a significant group effect, *post hoc* tests with Bonferroni adjustments were calculated as well. Furthermore, group × time pairwise comparisons were calculated in cases of a significant interaction effect to detect differences in the development of the two conditions.

#### Research Questions 3 and 4

To address the third research question, a hierarchical cluster analysis (Ward’s method with squared Euclidean distances) was conducted to find out whether there are specific subgroups of students that differ in using the standard written algorithm, the stepwise strategy, the compensation strategy, and the indirect addition adaptively at the points of measurement. The split strategy was again not part of the analysis since it could not have been used adaptively in the strategy test.

The cluster analysis detected four clusters since there was a comparatively big change regarding the distance coefficients between the four (224.02) and the three cluster solution (242.42). The results of the quality check of the cluster analysis were satisfying. Conformance checks with a hierarchical cluster analysis with Ward’s method and city-block distance (82.05%, κ = 0.74) as well as with K-means clustering as a confirmatory method (87.18%, κ = 0.82) showed a high validity of the allocation of the students to the clusters. Moreover, the clustering was examined with a discriminant analysis. The first discriminant function had a canonical correlation of 0.98 (eigenvalue = 20.15, explained variance = 84.24%, Wilk’s λ = 0.06, *p* < 0.001) and thus, contributed significantly to the separation of the groups, as well as the second function (eigenvalue = 2.61, explained variance = 10.93%, canonical correlation = 0.85, Wilk’s λ = 0.13, *p* < 0.001), and the third function (eigenvalue = 1.16, explained variance = 4.83%, canonical correlation = 0.73, Wilk’s λ = 0.46 *p* < 0.001). 97.44% of the original grouped cases and 94.87% of the cross-validated grouped cases were correctly classified. Table 5 shows the standardized canonical discriminant function coefficients for the three functions as well as the average discriminant coefficients to evaluate the discriminatory effect under consideration of all discrimination functions (Backhaus et al., 2000, p. 198). The variable compensation strategy at T3 has the biggest discriminatory effect for the first function, the variable indirect addition at T2 has the biggest effect for the second and the third function. On average, the variable indirect addition at T2 shows the greatest discriminatory effect. In addition to the quality check, we took the four cluster solution because of the good interpretability of the cluster profiles.

**Table 5:** Standardized canonical discriminant functions and average discriminant coefficients of the cluster solution.

	Discriminant coefficient			
Variable	Function 1	Function 2	Function 3	Average
Standard written algorithm T1	0.00	–0.06	0.04	–0.07
Standard written algorithm T2	0.07	0.04	–0.01	0.03
Standard written algorithm T3	0.11	0.14	–0.04	0.02
Standard written algorithm T4	0.10	0.05	–0.17	–0.04
Stepwise strategy T1	0.00	–0.06	–0.01	–0.02
Stepwise strategy T2	0.05	–0.03	0.41	0.14
Stepwise strategy T3	0.02	–0.04	0.34	0.11
Stepwise strategy T4	0.04	–0.07	0.37	0.13
Compensation strategy T1	0.03	0.07	–0.06	0.01
Compensation strategy T2	0.33	0.08	0.12	0.18
Compensation strategy T3	0.89	–0.15	–0.03	0.24
Compensation strategy T4	0.28	–0.02	0.04	0.10
Indirect addition T1	0.02	0.09	–0.05	0.02
Indirect addition T2	0.25	0.62	0.50	0.46
Indirect addition T3	0.21	0.30	0.13	0.21
Indirect addition T4	0.23	0.55	–0.30	0.16

To determine differences in the development of the strategy-specific adaptivity between the identified clusters,4 (group) × 4 (time) ANOVAs with repeated measures were conducted in consideration of all four points of measurement including *post hoc* tests (Bonferroni). Greenhouse–Geisser correction was used when the assumption of sphericity was violated. In cases of a significant group, time or interaction effect the same *post hoc* tests as already mentioned in the section above were calculated.

To address the fourth research question, to analyze in how far being part of a specific cluster depends on the prior arithmetical achievement and the teaching approach, a multinomial logistic regression was used, whereby the identified clusters were the dependent variable and the teaching condition as well as the prior arithmetical achievement the independent variables.

## Results

### Distribution of the Strategies – Flexibility

**FIGURE 5:**
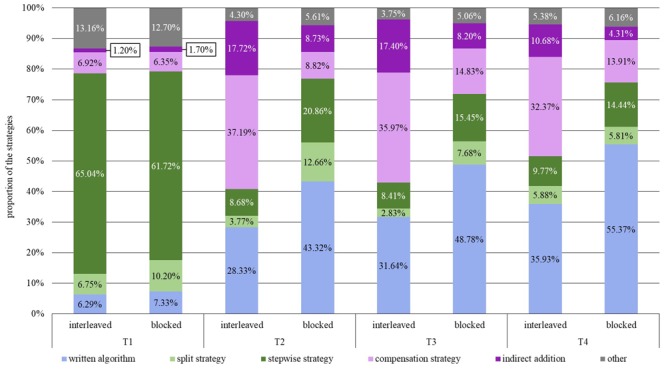
Distribution of the strategies used for solving the subtraction tasks.

To address the first research question, the strategy distributions of the two conditions were compared to establish whether the students of the interleaved practice use the subtraction strategies more flexibly after the treatment than the students of the blocked approach. Figure 5 gives an overview of the proportions of the use of the two shortcut strategies, i.e., the compensation strategy and the indirect addition (purple), the two decomposition strategies, i.e., the stepwise strategy and the split strategy (green), and the standard written algorithm (blue) for the interleaved and the blocked condition to solve three-digit subtraction problems at the four points of measurement.

A χ^2^-homogeneity test revealed just a marginally significant difference between the interleaved and the blocked group at T1 with a small effect size, χ^2^(5, *N* = 2288) = 10.55, *p* = 0.06, *C*_corr_ = 0.10. Thus, the proportion of the used strategies is only associated to a very limited extent with the teaching condition. As apparent from Figure 5, the students of the interleaved approach used the stepwise strategy slightly more often with a difference of 3.32%, whereas blocked approach students used the split strategy marginally more often with a difference of 3.45%. However, it can be assumed that these minor divergences at T1 between the groups do not affect the results for the measurement points after the treatment since the MANOVA in Section “Design and Participants” showed no significant difference between the two groups in how often the individual students applied the strategies at T1.

The two groups differed significantly at all points of measurement after the intervention, at T2, χ^2^(5, *N* = 2262) = 380.19, *p* < 0.001, *C*_corr_ = 0.54, T3, χ^2^(5, *N* = 2347) = 236.96, *p* < 0.001, *C*_corr_ = 0.43, and T4, χ^2^(5, *N* = 2344) = 176.44, *p* < 0.001, *C*_corr_ = 0.37, even though the effect decreased slightly over time. The students of the interleaved approach had a higher percentage in the application of the compensation strategy than the students of the blocked approach. Moreover, they used the indirect addition more often than the students of the blocked condition. Compared with this, the students of the blocked condition used the standard written algorithm more frequently than those of the interleaved condition, even though the use of the standard written algorithm increased in both conditions over time. While the compensation strategy was the most used strategy in the interleaved condition, the students of the blocked approach focused on the standard written algorithm after its introduction. The second most commonly used strategy in the blocked condition was the stepwise strategy, whereas this strategy had rarely been applied by the students of the interleaved practice after the intervention (T2–T4). Regarding the split strategy, the students of the blocked condition used it on T2 and T3 more often than those of the interleaved approach. On T4, the percentages regarding the use of the split strategy were almost equal in the two conditions.

In summary, the students of the interleaved practice showed a higher percentage in the use of the compensation strategy and the indirect addition, whereas the students of the blocked condition used the standard written algorithm and the stepwise strategy more frequently.

### Strategy-Specific Adaptivity

The results of the strategy distributions show that the students of the interleaved approach used the two shortcut strategies more often and the standard written algorithm as well as the stepwise strategy less often than the students of the blocked condition. However, these results do not implicate how much more adaptively the strategies were used. The second research question investigates whether the two conditions differ in their strategy-specific adaptivity. Table 6 shows the means and standard deviations of the relative adaptive use of the standard written algorithm, the stepwise strategy, the compensation strategy, and the indirect addition at the four points of measurement for the interleaved and blocked condition as well as the results of the *post hoc* comparisons in cases of a significant group effect. The split strategy was not part of the analysis since it could not have been used adaptively (see the section “Flexibility and Strategy-Specific Adaptivity”). For instance, the students of the interleaved condition used the standard written algorithm in 38.13% (*SD* = 34.19%) of the time adaptively 1 day after the intervention (T2), and thus, significantly more adaptive than the students of the blocked approach (*M* = 21.72%, *SD* = 25.31%).

**Table 6:** Means and standard deviations of the strategy-specific adaptivity at T1, T2, T3, and T4 and results of the *post hoc* comparisons (group effect).

	Interleaved			Blocked			
	*n*	*M*	*SD*	*n*	*M*	*SD*	*Post hoc* comparisons
Standard written algorithm T1	113	3.08%	12.22%	109	4.08%	14.71%	
Standard written algorithm T2	112	38.13%	34.19%	110	21.72%	25.31%	interleaved > blocked
Standard written algorithm T3	116	60.69%	36.04%	113	41.94%	33.10%	interleaved > blocked
Standard written algorithm T4	115	53.37%	36.62%	111	40.64%	29.03%	interleaved > blocked
Stepwise strategy T1	113	30.77%	24.73%	109	30.66%	25.69%	
Stepwise strategy T2	112	27.35%	42.65%	110	27.33%	36.35%	
Stepwise strategy T3	116	24.06%	41.04%	113	19.86%	34.26%	
Stepwise strategy T4	115	16.92%	33.50%	111	15.11%	29.46%	
Compensation strategy T1	113	6.41%	20.22%	109	7.15%	21.39%	
Compensation strategy T2	112	64.96%	36.88%	110	20.48%	37.43%	interleaved > blocked
Compensation strategy T3	116	64.20%	37.66%	113	30.11%	41.52%	interleaved > blocked
Compensation strategy T4	115	55.93%	39.19%	111	20.97%	35.39%	interleaved > blocked
Indirect addition T1	113	2.65%	16.15%	109	3.59%	17.42%	
Indirect addition T2	112	63.03%	47.25%	110	22.54%	40.46%	interleaved > blocked
Indirect addition T3	116	63.15%	47.51%	113	25.20%	42.39%	interleaved > blocked
Indirect addition T4	115	40.00%	49.20%	111	15.24%	36.02%	interleaved > blocked

*In the column “*post hoc* comparisons” significant group effects are shown for each point of measurement and each strategy represented by “>”, which also indicates which group was superior.*

ANOVAs with repeated measures revealed that the students of the interleaved approach had an advantage regarding the adaptive use of the *standard written algorithm, F*(1,193) = 25.62, *p* < 0.001, $\eta_{p}^{2}$ = 0.12. There was a main effect of time, *F*(3,579) = 149.56, *p* < 0.001, $\eta_{p}^{2}$ = 0.44, with pairwise comparisons revealing significant increases between T1 and T2 (*p* < 0.001, *d* = 0.79), T1 and T3 (*p* < 0.001, *d* = 1.37), T1 and T4 (*p* < 0.001, *d* = 1.25), T2 and T3 (*p* < 0.001, *d* = 0.64), and T2 and T4 (*p* < 0.001, *d* = 0.43). A small interaction effect of time and group was found, *F*(3,579) = 25.62, *p* < 0.001, $\eta_{p}^{2}$ = 0.04. Pairwise comparisons showed that both groups improved significantly from T1 to T2 (blocked: *p* < 0.001, *d* = 0.60; interleaved: *p* < 0.001, *d* = 1.00), from T1 to T3 (blocked: *p* < 0.001, *d* = 1.20; interleaved: *p* < 0.001, *d* = 1.74), and from T1 to T4 (blocked: *p* < 0.001, *d* = 1.07; interleaved: *p* < 0.001, *d* = 1.37). Even after the intervention, both groups improved in the adaptive application of the standard written algorithm from T2 to T3 (blocked: *p* < 0.001, *d* = 0.67; *p* < 0.001, *d* = 0.63), and from T2 to T4 (blocked: *p* < 0.001, *d* = 0.60; interleaved: *p* = 0.001, *d* = 0.33). There was no significant difference between T3 and T4 for the blocked group, while the adaptive use of the standard written algorithm of the students of the interleaved approach decreased significantly with a small effect (*p* = 0.02, *d* = -0.26).

Regarding the *stepwise strategy*, there was only a significant time effect, *F*(2.88,555.96) = 9.94, *p* < 0.001, $\eta_{p}^{2}$ = 0.05, showing significant decreases between T1 and T3 (*p* = 0.02, *d* = -0.22), T1 and T4 (*p* < 0.001, *d* = -0.40), and T2 and T4 (*p* < 0.001, *d* = -0.30). Unexpectedly, no significant group effect, *F*(1,193) = 0.13, *p* = 0.72, $\eta_{p}^{2}$ = 0.00, and no interaction effect of group and time, *F*(2.88,555.96) = 0.13, *p* = 0.94, $\eta_{p}^{2}$ = 0.00, was found, indicating that the adaptive use of the stepwise strategy deteriorated over time in both groups equally.

The students of the interleaved condition were superior in the adaptive use of the *compensation strategy* with a strong group effect, *F*(1,193) = 58.27, *p* < 0.001, $\eta_{p}^{2}$ = 0.23. There was a significant effect of time, *F*(2.49,479.78) = 109.51, *p* < 0.001, $\eta_{p}^{2}$ = 0.36, with significant increases between T1 and T2 (*p* < 0.001, *d* = 0.84), T1 and T3 (*p* < 0.001, *d* = 0.92), and T1 and T4 (*p* < 0.001, *d* = 0.76), and a significant decrease between T3 and T4 (*p* < 0.001, *d* = -0.36). Moreover, a significant interaction effect of group and time was found, *F*(2.49,479.78) = 35.78, *p* < 0.001, $\eta_{p}^{2}$ = 0.16. As *post hoc* tests showed, the strategy-specific adaptivity of the compensation strategy increased in both groups between T1 and T2 (blocked: *p* = 0.008, *d* = 0.33; interleaved: *p* < 0.001, *d* = 1.62), T1 and T3 (blocked: *p* < 0.001, *d* = 0.48; interleaved: *p* < 0.001, *d* = 1.56), T1 and T4 (blocked: *p* = 0.02, *d* = 0.32; interleaved: *p* < 0.001, *d* = 1.32), and deteriorated in both groups between T3 and T4 (blocked: *p* = 0.02, *d* = -0.21; interleaved: *p* = 0.001, *d* = -0.23). An increase between T2 and T3 was only found for the blocked approach (*p* = 0.05, *d* = 0.19).

Furthermore, there were significant differences between the conditions regarding the strategy-specific adaptivity of the *indirect addition* with advantage for the interleaved condition, *F*(1,193) = 39.27, *p* < 0.001, $\eta_{p}^{2}$ = 0.17. A significant effect of time was detected, *F*(2.83,545.74) = 76.35, *p* < 0.001, $\eta_{p}^{2}$ = 0.28, with significant increases between T1 and T2 (*p* < 0.001, *d* = 0.79), T1 and T3 (*p* < 0.001, *d* = 0.85), and T1 and T4 (*p* < 0.001, *d* = 0.56). The adaptive use of the indirect addition decreased significantly between T2 and T4 (*p* < 0.001, *d* = -0.34), and T3 and T4 (*p* < 0.001, *d* = -0.39). There was also an interaction effect of group and time, *F*(2.83,545.74) = 20.21, *p* < 0.001, $\eta_{p}^{2}$ = 0.10. *Post hoc* tests showed that the students of both groups increased significantly between T1 and T2 (blocked: *p* = 0.001, *d* = 0.44; interleaved: *p* < 0.001, *d* = 1.19), and T1 and T3 (blocked: *p* < 0.001, *d* = 0.47; interleaved: *p* < 0.001, *d* = 1.34). A significant increase between T1 and T4 (*p* < 0.001, *d* = 0.77), and a significant decrease between T2 and T4 (*p* < 0.001, *d* = -0.63), and T3 and T4 (*p* < 0.001, *d* = -0.67) was only found for the interleaved condition.

Summarizing the results, the students of the interleaved practice showed a higher strategy-specific adaptivity at T2, T3, and T4 regarding the standard written algorithm, the compensation strategy, and the indirect addition, while both conditions had the same low level in the strategy-specific adaptivity of the stepwise strategy.

### Cluster Analysis

**FIGURE 6:**
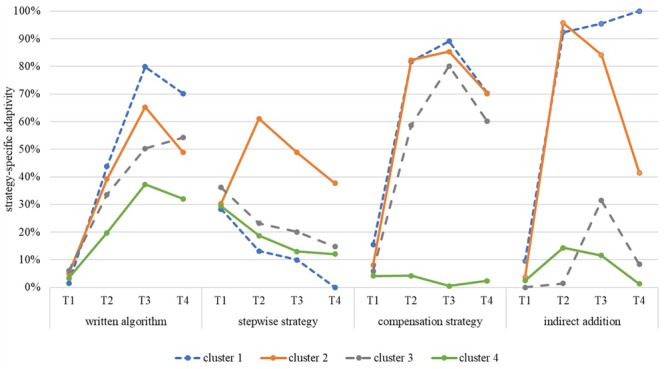
Result of the cluster analysis.

The goal of the third research question was to detect different adaptivity profiles capturing variability between and within the students to ascertain whether clusters of students can be determined that differed in their adaptive use of the standard written algorithm, the stepwise strategy, the compensation strategy, and the indirect addition. The split strategy was again not part of the analysis since it could not have been used adaptively (see the section “Flexibility and Strategy-Specific Adaptivity”). A hierarchical cluster analysis revealed four subgroups of students varying in their degree of strategy-specific adaptivity. As Figure 6 illustrates, cluster 1 (18.46%) consisted of students with a relatively high level of adaptivity in all strategies, except for the stepwise strategy. Cluster 2 (21.03%) grouped those students together with a comparatively high strategy-specific adaptivity of all strategies, whereas cluster 3 (17.95%) consisted of students with a low level of adaptivity concerning the stepwise strategy and the indirect addition, and a comparatively high level in the adaptive use of the standard written algorithm and the compensation strategy. Finally, the fourth cluster (42.56%) grouped together those students characterized by a comparatively non-adaptive use of all four strategies.

In Table 7, the exact means and standard deviations as well as the *post hoc* comparisons of the group effects of the strategy-specific adaptivity of the standard written algorithm, the stepwise strategy, the compensation strategy, and the indirect addition at T1, T2, T3, and T4 are shown for the four clusters. For instance, the students of cluster 1 (*M* = 43.72%, *SD* = 32.62%) and cluster 2 (*M* = 39.22%, *SD* = 38.33%) used the standard written algorithm significantly more adaptively at T2 than cluster 4 (*M* = 19.69%, *SD* = 22.28%), whereas cluster 3 (*M* = 33.58%, *SD* = 29.89%) did not differ significantly from the other three clusters.

**Table 7:** Means and standard deviations of the strategy-specific adaptivity at T1, T2, T3, and T4 for the four clusters and results of the *post hoc* comparisons (group effect).

	Cluster 1 (*n* = 36)		Cluster 2 (*n* = 41)		Cluster 3 (*n* = 35)		Cluster 4 (*n* = 83)		
Variable	*M*	*SD*	*M*	*SD*	*M*	*SD*	*M*	*SD*	*Post hoc* comparisons
Standard written algorithm T1	1.39%	8.33%	4.96%	15.54%	5.98%	16.91%	3.34%	13.59%	
Standard written algorithm T2	43.72%	32.62%	39.22%	38.33%	33.58%	29.89%	19.69%	22.28%	1, 2 > 4
Standard written algorithm T3	79.86%	24.88%	65.27%	36.77%	50.26%	37.91%	37.24%	27.85%	1 > 3, 4; 2 > 4
Standard written algorithm T4	70.13%	27.40%	48.91%	37.86%	54.19%	32.49%	32.01%	26.53%	1, 2, 3 > 4
Stepwise strategy T1	28.32%	23.27%	30.31%	23.76%	36.18%	28.69%	29.44%	25.82%	
Stepwise strategy T2	13.13%	33.42%	61.03%	47.49%	23.13%	38.06%	18.68%	29.63%	2 > 1, 3, 4
Stepwise strategy T3	9.91%	28.71%	48.78%	50.61%	20.05%	37.70%	12.99%	26.09%	2 > 1, 3, 4
Stepwise strategy T4	0.00%	0.00%	37.71%	46.49%	14.74%	30.40%	12.07%	23.44%	2 > 1, 3, 4
Compensation strategy T1	15.43%	31.08%	8.05%	21.50%	5.83%	14.92%	4.13%	18.74%	
Compensation strategy T2	81.78%	26.68%	82.28%	21.67%	58.69%	35.44%	4.15%	18.82%	1, 2 > 3; 1, 2, 3 > 4
Compensation strategy T3	89.13%	11.70%	85.38%	17.85%	80.08%	8.30%	0.47%	4.27%	1, 2 > 3; 1, 2, 3 > 4
Compensation strategy T4	70.36%	35.40%	70.18%	29.27%	60.15%	34.04%	2.29%	11.97%	2 > 3; 1, 2, 3 > 4
Indirect addition T1	9.60%	28.67%	3.55%	17.00%	0.00%	0.00%	2.41%	15.43%	
Indirect addition T2	92.32%	24.54%	95.72%	17.74%	1.43%	8.45%	14.32%	33.94%	1, 2 > 3, 4
Indirect addition T3	95.52%	17.68%	84.15%	36.12%	31.43%	47.10%	11.56%	30.93%	1, 2 > 3, 4
Indirect addition T4	100.00%	0.00%	41.46%	49.88%	8.35%	27.68%	1.20%	10.98%	1 > 2; 1, 2 > 3, 4

*In the column “*post hoc* comparisons” significant group effects are shown for each point of measurement and each strategy represented by “>”, which also indicates which group was superior.*

ANOVAs with repeated measures including *post hoc* tests were conducted to reveal in which strategies and at which points of measurement the four clusters differed significantly. Regarding the *standard written algorithm*, a significant effect of group was found, *F*(3,191) = 21.20, *p* < 0.001, $\eta_{p}^{2}$ = 0.25. Furthermore, there was a significant effect of time, *F*(3,573) = 170.46, *p* < 0.001, $\eta_{p}^{2}$ = 0.47, with significant increases between T1 and T2 (*p* < 0.001, *d* = 0.79), T1 and T3 (*p* < 0.001, *d* = 1.37), T1 and T4 (*p* < 0.001, *d* = 1.25), T2 and T3 (*p* < 0.001, *d* = 0.64), and T2 and T4 (*p* < 0.001, *d* = 0.43). Furthermore, the clusters differed in their development of their strategy-specific adaptivity of the standard written algorithm as the significant interaction effect of time and group (cluster) showed, *F*(9,573) = 6.77, *p* < 0.001, $\eta_{p}^{2}$ = 0.10.

*Post hoc* comparisons were calculated to detect the differences in the development of the four clusters. In Table 8, the results of those *post hoc* comparisons, i.e., the developments between the points of measurement for each cluster separately, are shown for the standard written algorithm and the other subtraction strategies. Cluster 1 showed the biggest increase after the intervention in using the standard written algorithm adaptively – shortly after the intervention and in the long-term. But the three other clusters did also develop a higher level in the adaptive application of this strategy compared to T1. Cluster 2 was the only group showing a significant decrease between T3 and T4 in using the standard written algorithm adaptively – the other clusters benefitted sustainably.

**Table 8:** Results of the *post hoc* comparisons for the interaction of cluster and time.

		Cluster 1	Cluster 2	Cluster 3	Cluster 4
	*Post hoc*
	comparisons	*d*	*d*	*d*	*d*
Standard written algorithm			
	T1 – T2	1.13***	0.76***	0.82***	0.71***
	T1 – T3	2.75***	1.45***	1.22***	1.23***
	T1 – T4	2.39***	1.13***	1.47***	1.08***
	T2 – T3	0.98***	0.52***	0.47*	0.71**
	T2 – T4	0.92***		0.62**	0.42*
	T3 – T4		–0.35*		
Stepwise strategy			
	T1 – T2		0.65***		
	T1 – T3		0.34*		–0.48**
	T1 – T4	–1.22***		–0.54**	–0.56***
	T2 – T3				
	T2 – T4		–0.42***		
	T3 – T4				
Compensation strategy			
	T1 – T2	1.64***	2.66***	1.57***	
	T1 – T3	2.25***	2.85***	4.25***	
	T1 – T4	1.20***	1.83***	1.61***	
	T2 – T3		0.62***		
	T2 – T4				
	T3 – T4	–0.53***	–0.66***	–0.55***	
Indirect addition			
	T1 – T2	1.79***	3.85***		0.31**
	T1 – T3	2.67***	2.12***	0.67***	
	T1 – T4	3.15***	0.70***		
	T2 – T3			0.59***	
	T2 – T4		–0.96***		–0.43***
	T3 – T4		–0.76***	–0.46***	

*^∗^*p* < 0.05, ^∗∗^*p* < 0.01, ^∗∗∗^*p <* 0.001.*

Concerning the *stepwise strategy*, the clusters differed significantly in the adaptive use, *F*(3,191) = 19.96, *p* < 0.001, $\eta_{p}^{2}$ = 0.24. There also was a significant main effect of time, *F*(3,573) = 9.65, *p* < 0.001, $\eta_{p}^{2}$ = 0.05, with significant decreases between T1 and T4 (*p* < 0.001, *d* = -0.40), and T2 and T4 (*p* < 0.001, *d* = -0.30). Moreover, there was an interaction effect between group and time, *F*(9,573) = 4.95, *p* < 0.001, $\eta_{p}^{2}$ = 0.07, indicating different developments of the clusters in the adaptive use of the stepwise strategy. The students of cluster 1, cluster 3, and cluster 4 deteriorated significantly between T1 and T4, while only the students of cluster 2 showed an increase in the adaptive use of the stepwise strategy between T1 and T2, and T1 and T3, and a significant decrease between T2 and T4.

For the *compensation strategy*, there was a strong main effect of group, *F*(3,191) = 347.45, *p* < 0.001, $\eta_{p}^{2}$ = 0.85. There was a strong and significant effect of time, *F*(2.69,513.83) = 254.63, *p* < 0.001, $\eta_{p}^{2}$ = 0.57. A *post hoc* test revealed significant increases between T1 and T2 (*p* < 0.001, *d* = 0.84), T1 and T3 (*p* < 0.001, *d* = 0.92), T1 and T4 (*p* < 0.001, *d* = 0.76), and T2 and T3 (*p* = 0.004, *d* = 0.15), and a significant decrease between T3 and T4 (*p* < 0.001, *d* = -0.33). A significant and strong interaction effect of group and time was found, *F*(8.07,513.83) = 254.63, *p* < 0.001, $\eta_{p}^{2}$ = 0.42. Thus, the clusters developed differently over time concerning the adaptive use of the compensation strategy. While cluster 1, cluster 2, and cluster 3 developed almost equally with significant increases until T3 and a significant decrease from T3 to T4, the students of cluster 4 did not show any significant differences in the adaptive use of the compensation strategy between any points of measurement. Their strategy-specific adaptivity stayed stable at a low level.

Concerning the *indirect addition*, the four clusters differed significantly in their strategy-specific adaptivity, *F*(3,191) = 218.61, *p* < 0.001, $\eta_{p}^{2}$ = 0.77. There was a significant time effect, *F*(2.83,540.21) = 149.06, *p* < 0.001, $\eta_{p}^{2}$ = 0.44, with significant increases between T1 and T2 (*p* < 0.001, *d* = 0.79), T1 and T3 (*p* < 0.001, *d* = 0.85), and T1 and T4 (*p* < 0.001, *d* = 0.56), and significant decreases between T2 and T4 (*p* < 0.001, *d* = -0.34) as well as between T3 and T4 (*p* < 0.001, *d* = -0.41). The four clusters differed significantly and strongly in their development concerning the adaptive use of the indirect addition, *F*(8.48,540.21) = 40.88, *p* < 0.001, $\eta_{p}^{2}$ = 0.39. Cluster 1 was the only group showing no decreases over the four points of measurement. The students of this group had very strong increases in using the indirect addition adaptively and they maintained their learning success. The students of cluster 2 also had an equally high increase between T1 and T2, T1 and T3, and T1 and T4 in using the indirect addition adaptively. However, they deteriorated significantly between T2 and T4, and T3 and T4. Cluster 3 and cluster 4 increased their strategy-specific adaptivity briefly, but deteriorated afterward so that their adaptive use of the indirect addition at T4 was at the same level as it was before the intervention.

Summarizing the results, four clusters were detected differing in their strategy-specific adaptivity of the subtraction strategies. Cluster 2 grouped those students together with a comparatively high adaptivity in the use of all subtraction strategies. In comparison, students in cluster 1 showed a high level of adaptive strategy use in all strategies except for the stepwise strategy and cluster 3 is characterized by a strategy-specific adaptivity which is limited to the written algorithm and the compensation strategy. The advantage of the strategy-specific adaptivity of cluster 1 (except the stepwise strategy) and cluster 2 could be shown for all points of measurement after the treatment. Finally, the students of cluster 4 had a comparatively low strategy-specific adaptivity of all strategies at all points of measurement.

### Influence of Prior Knowledge and Treatment on the Cluster Membership

Based on the four clusters, the fourth research question explored whether belonging to a specific cluster depends on the teaching approach and the prior arithmetical achievement. A descriptive view on the distribution of the students of the two conditions to the clusters showed that the students of the interleaved approach were the predominant part of cluster 1 (interleaved: *n* = 27, blocked: *n* = 9) and cluster 2 (interleaved: *n* = 33, blocked: *n* = 8), i.e., the clusters with a high strategy-specific adaptivity in (almost) all subtraction strategies. By contrast, the students of the blocked approach were more often grouped in cluster 4 (interleaved: *n* = 21, blocked: *n* = 62), which was the cluster with the lowest adaptive use of the strategies. On the other hand, the students of both conditions were almost equally distributed in cluster 3 (*n* = interleaved: 20, blocked: *n* = 15), i.e., the cluster with a high level of adaptivity regarding the standard written algorithm and the compensation strategy, but a comparatively low level regarding the stepwise strategy and the indirect addition. Cluster 1 had an average of 14.44 (*SD* = 5.09) points in the arithmetical achievement test at T0. Cluster 2 reached 14.75 (*SD* = 5.93) and cluster 3 12.88 (*SD* = 5.36) points on average, while the students of cluster 4 had a lower prior achievement in arithmetic (*M* = 9.03, *SD* = 5.05).

A subsequent multinomial logistic regression with cluster 4 as reference category supported the descriptive findings. The model fit, χ^2^(6) = 90.79, *p* < 0.001, as well as the Deviance Goodness-of-Fit measure, χ^2^(138) = 112.85, *p* = 0.94, indicate that the multinomial logit model is satisfactory. Moreover, the likelihood ratio tests for the independent variables treatment, χ^2^(3) = 51.96, *p* < 0.001, and arithmetical achievement, χ^2^(3) = 48.14, *p* < 0.001, show a satisfactory fit of the model as well, which is supported by a relatively high Pseudo *R*^2^ (Cox and Snell = 0.39, Nagelkerke = 0.42, McFadden = 0.19). 51.61% of the cases were correctly classified. The results of the multinomial logistic regression are shown in Table 9.

**Table 9:** Multinomial logistic regression predicting the affiliation to a specific cluster (reference category: cluster 4).

Dependent variable	Independent variable	*B*	*SE*	Wald	*Odds ratio*	*p*
Cluster 1	Treatment (reference category: blocked)	2.89	0.57	25.69	17.75	<0.001
	Arithmetical achievement (T0) (z-score)	0.25	0.05	24.73	4.21	<0.001
Cluster 2	Treatment (reference category: blocked)	3.13	0.57	30.33	22.89	<0.001
	Arithmetical achievement (T0) (z-score)	0.26	0.05	28.47	4.61	<0.001
Cluster 3	Treatment (reference category: blocked)	1.70	0.48	12.33	5.46	<0.001
	Arithmetical achievement (T0) (z-score)	0.17	0.04	14.58	2.67	<0.001

The results reveal that the students of the interleaved practice had a 17.75 times higher chance of belonging to cluster 1 with reference to cluster 4. The likelihood of being in cluster 1 increased by 4.21 times when having an arithmetical achievement of one standard deviation above the total mean. As a result, the independent variable treatment makes a much greater contribution for predicting the affiliation to cluster 1 than the prior arithmetical achievement at T0. Regarding cluster 2 with reference to cluster 4, the *odds ratio* shows that the probability of being in cluster 2 rises significantly by 22.89 times when being taught interleaved. In comparison to the probability of being in cluster 1, the arithmetical achievement had a much smaller effect (*odds ratio* = 4.61). For the likelihood of being in cluster 3, being taught interleaved had a smaller, but still substantial effect (*odds ratio* = 5.46), while the arithmetical achievement again had a smaller effect (*odds ratio* = 2.67).

Summarizing the results, the cluster membership was strongly related to the teaching approach: Being taught interleaved was a strong predictor for the affiliation to clusters with a higher strategy-specific adaptivity in all/some strategies with reference to a cluster with a comparatively non-adaptive use of all strategies. The prior arithmetical achievement had a much smaller influence than the teaching approach.

## Discussion

The results of this study suggest that an interleaved approach extended by prompts to compare (1) is practicable and can be well integrated into regular elementary school classrooms. Moreover, (2) it enhances the flexible and adaptive use of subtraction strategies among third graders compared to a blocked approach with prompts for within-comparisons. The analysis of the strategy distributions showed a lower level of flexibility in the blocked condition: The students of the blocked approach predominantly used the standard written algorithm after its introduction to solve subtraction tasks, whereas the compensation strategy and the indirect addition were used comparatively rarely. The dominance of the standard written algorithm even increased over time. As a result, our study replicates the findings of previous research regarding the dominance of the standard written algorithm after its introduction (Selter, 2001; Torbeyns and Verschaffel, 2016; Heinze et al., 2018). Compared to this, the students of the interleaved condition used the compensation strategy and the indirect addition relatively often, also after the introduction of the standard written algorithm, even though there was a small increase of the use of the standard written algorithm over time as well. Still, these results indicate that interleaving subtraction strategies can lead to some kind of resilience against using the standard written algorithm. Furthermore, it can lead to a higher level of a flexible application of number-based strategies as well as the standard written algorithm to solve three-digit subtraction tasks. Regarding this, it should be noted that no absolute statement about the typical proportion of strategy use of third graders can be made. Since the strategy test evoked the strategies in different quantities, only a comparison between the two groups and their development between the four points of measurement is possible. As already mentioned in Section “Flexibility and Strategy-Specific Adaptivity”, the utilized strategy test triggered the use of the compensation strategy and the indirect addition the most, so that the strategy distribution is not balanced. This was due to the methodical decision to focus on the shortcut strategies (compensation strategy and indirect addition) as subtraction strategies which are rarely applied by elementary school students, because they tend to focus more on the stepwise strategy and the standard written algorithm after its introduction. However, the students of both conditions used an equal amount of time for a specific strategy, whereas the time percentages between the different strategies differed. Therefore, these results may indicate that the students of the interleaved approach consider task characteristics before choosing a strategy leading to a more adaptive strategy use, which was shown by the subsequent analysis. In consideration of the number of tasks triggering the indirect addition, this strategy was used relatively rarely in both conditions, even though it had been used more frequently in the interleaved approach. This may be due to different task characteristics that evoke the two shortcut strategies: While they are comparatively obvious for the compensation strategy (the subtrahend is close to a full hundred) so that only the subtrahend has to be taken into consideration, students have to take the relation of the minuend and the subtrahend into account when deciding if the indirect addition is efficient for a specific subtraction task (e.g., 502 – 498: A superficial look at this task might evoke using the compensation strategy. Only when both numbers are considered, it does become apparent that the indirect addition is more adaptive since only one solution step is necessary). Moreover, it might be counter-intuitive for students to solve subtraction tasks by addition (De Smedt et al., 2010). Hence, the acquisition of the adaptive application of the indirect addition might be more challenging. Nevertheless, the students of the interleaved condition used the indirect addition more often, which could be due to the fact that they explicitly compared the two shortcut strategies (between-comparison) in the teaching unit and therefore, are superior in discriminating tasks that evoke those two strategies.

The students of the interleaved condition showed not only a higher level of flexibility but also a higher level of strategy-specific adaptivity of almost all subtraction strategies. The only strategy in which the students of the interleaved condition were not superior was the stepwise strategy. This could be explained by the characteristics of the stepwise strategy itself: While the use of the compensation strategy and the indirect addition is predestined for specific types of subtraction tasks that are comparatively easy to identify, there are no explicit task characteristics showing that the stepwise strategy is adaptive – instead it is more a procedure of exclusion in consideration of the other strategies (e.g., 354 – 227: There is not a small difference between the minuend and the subtrahend, the subtrahend is not close to a full hundred, and two digits of the subtrahend are bigger than those of the minuend; ergo the indirect addition, the compensation strategy, and the split strategy are not adaptive, while the stepwise strategy and the standard written algorithm are adaptive). The students of both conditions might have used the stepwise strategy only if they have ruled out the other strategies erroneously leading to a comparatively non-adaptive use. Since the students of the interleaved practice did not use the stepwise strategy very often, it may be the case that this strategy was only then applied if the students did not know which of the other strategies would have been adaptive and therefore, they did not use it efficiently. Regarding the adaptive use of the standard written algorithm, the students of the interleaved condition benefitted significantly at all points of measurement after the intervention. This result supports the assumption of the standard written algorithm-resilience that can be caused by interleaving subtraction strategies. Moreover, the students of the interleaved condition showed a higher level of adaptive use of the compensation strategy and the indirect addition. For both subtraction strategies the effects were even more substantial than for the standard written algorithm. However, there was a huge decrease of the effect over time, especially for the indirect addition. Since there was a decrease of the adaptive use over time of not only the indirect addition but all subtraction strategies, it seems advisable to integrate additional booster sessions refreshing the students’ knowledge of the adaptive application of the strategies.

Starting from a person-centered view, a subsequent hierarchical cluster analysis revealed four different subgroups of students differing in their adaptive use of the stepwise strategy, the compensation strategy, the indirect addition, and the standard written algorithm. A multinomial logistic regression with cluster 4, i.e., the cluster with a low strategy-specific adaptivity regarding all strategies, as reference category revealed that being part of the others was positively related to (1) the treatment, with interleaving having a positive impact, and (2) the prior arithmetical achievement. For all clusters the teaching approach was the major predictor. Especially for cluster 1 grouping students together with a high level of adaptivity regarding all strategies except for the stepwise strategy and cluster 2, i.e., the cluster characterized by a high strategy-specific adaptivity in all subtraction strategies, the probability of the affiliation to these clusters was highly related to the teaching approach.

Summarizing the results, interleaving subtraction strategies with supporting discrimination processes by prompts to compare seems to foster the flexible strategy use and the ability to choose an appropriate strategy based on specific tasks and their characteristics sustainably. Therefore, this study supplements previous research on interleaved practice in mathematics, which did not thoroughly show positive effects (Brunmair and Richter, 2019). Both, interleaving as well as including comparisons in students’ learning, are considered to be desirable difficulties for enhancing long-term retention (Holyoak, 2005; Dunlosky et al., 2013). The impressive effect on the flexible and adaptive strategy choice of elementary school students found in our study may be explained by the comparison processes triggered by the interleaved structure of the teaching unit that were supported by prompts to compare the subtraction strategies. These multiple comparisons may demand a higher cognitive effort from the students, since these students have to deal with various learning contents at once, while students in a blocked learning approach focus on one category. Still, comparisons provide the advantage of getting students to reflect their strategy choice for every subtraction task. Thus, interleaved practice with comparison processes supported by prompts can help students to discriminate between the subtraction strategies and can lead to a more flexible and adaptive use. In blocked learning of subtraction strategies, students do not have to discriminate the strategies which explains our results in favor of the interleaved condition. Although our results show a clear advantage of interleaving subtraction strategies including prompts to compare, it should be noted that we combined interleaved practice with comparisons. Consequently, a final statement about which of the two desirable difficulties (interleaving or comparing) led to the better learning outcomes of the students of the interleaved condition cannot be made but has to be evaluated in further studies.

As stated, interleaved practice may require a higher cognitive effort from the students. Hence, further research should investigate whether all students benefit equally from interleaving subtraction strategies. On the one hand, it is conceivable that the positive impact of interleaving subtraction strategies is affected by the arithmetical achievement since multiple comparisons can cause a cognitive overload for students with a low prior knowledge (Chandler and Sweller, 1991; Sweller and Chandler, 1994). Previous research has shown inconsistent results regarding the importance of previous knowledge for the effectivity of contrast and discrimination processes (for an overview, see Guo et al., 2012). For instance, Rittle-Johnson et al. (2009) demonstrated in their study that students with a lower prior knowledge benefitted more when they studied algebra examples sequentially or compared problem types that were solved in the same way. Comparing methods had a negative impact on the learning outcomes in the posttest for these students; however, students with a higher prior knowledge profited from comparing methods. In the studies of both Durkin and Rittle-Johnson (2012) and Ziegler and Stern (2014), the effect of comparing in mathematics was not moderated by the prior knowledge of the students. One reason for these differing results regarding the relevance of prior knowledge on the effectivity of comparisons in learning might be the concrete implementation. Rittle-Johnson et al. (2012) revealed in a replication of their already mentioned study (Rittle-Johnson et al., 2009) that students with a lower prior knowledge benefitted just as much as those with a higher prior knowledge from comparing when more possibilities to practice were provided and the pace of instruction was decelerated. On the other hand, motivational variables (e.g., attitude, goal orientations, self-efficacy) and the cognitive motivation of students (need for cognition), i.e., the enjoyment of being involved in cognitive activities, seem to be dispositions of students that could moderate the effect of classroom instructions (e.g., Ackerman and Heggestad, 1997; Preckel et al., 2006; Dalbert and Radant, 2008; Hughes et al., 2013; Preckel, 2014; Luong et al., 2017). The effect of these variables might be even more substantial for desirably difficult classroom instructions since they hamper learning in the short-term and therefore, require a higher cognitive effort from the individuals before learning successes occur. Previous studies have not yet investigated, if the mentioned motivational and cognitive dispositions of students moderate the effect of interleaved practice in elementary school mathematics, so that further research is required.

Furthermore, it has to be taken into consideration that we took a normative perspective when rating the adaptivity of strategy use which is partially criticized in the literature (Threlfall, 2002; Verschaffel et al., 2009). For a comprehensive evaluation of adaptivity, the prerequisites as well as the social context seem to be essential as well. While the social context may play a minor role in our study, since the introduction of the strategies and the teacher behavior were standardized by a script, the prerequisites of students may have a greater impact on adaptivity. For students with a low previous knowledge it might be less error-prone to use, for instance, the standard written algorithm consistently since they need less knowledge about number relations to apply this strategy. A method that would make the integration of a subjective perspective on adaptivity possible is the choice-/no-choice method (Siegler and Lemaire, 1997) that has already been successfully applied in numerous studies (e.g., Torbeyns et al., 2005, 2009a,b; Torbeyns and Verschaffel, 2016). However, this method takes the speed and accuracy into account when assessing adaptivity, whereas we had a narrower definition of adaptivity in our study. Moreover, this method limits the demonstration of the strategy repertoire since the students need to solve tasks with previously selected subtraction strategies in the no-choice condition. By contrast, an open strategy test as used in our study has the advantage of measuring a wide range of different subtraction strategies. Nonetheless, the choice-/no-choice method is a promising approach for further research to assess another facet of the adaptive use of subtraction strategies including a subjective perspective.

## Conclusion

This study demonstrated that interleaved practice including explicit prompts to compare can foster the flexible and adaptive application of subtraction strategies as high-similarity categories by third graders. However, further research should explore whether these positive findings are transferable to (1) other mathematical contents, (2) other school subjects, and (3) whether elementary school students also benefit from interleaving low-similarity categories as the study by Rohrer et al. (2014) showed for seventh graders.

## Ethics Statement

This study was carried out in accordance with the recommendations of the Declaration of Helsinki as well as the ethical guidelines of the German Psychologists Association (BDP) and the German Psychological Society (DGP). The protocol was approved by the Ethics Committee of the Faculty of Human Sciences (University of Kassel). All parents gave written informed consent in accordance with the Declaration of Helsinki.

## Author Contributions

FL supervised the project. KW, JA, and FL conceived and planned the experimental study. KW and LN were part of the teacher-team. KW, JA, and LN performed parts of the measurements. SV had the idea to investigate the effectivity of interleaved practice for each subtraction strategy. LN performed the calculations and drafted the following parts of the manuscript: Introduction, Materials and Methods (Design and Participants, Instruments: Calculation of the Strategy-Specific Adaptivity, Analysis), Results, and Discussion. KW drafted the other parts of the section ‘Materials and Methods.’ KW, JA, SV, and FL peer reviewed the manuscript critically. All authors approved the article for publication.

## Conflict of Interest Statement

The authors declare that the research was conducted in the absence of any commercial or financial relationships that could be construed as a potential conflict of interest.

## Footnotes

1 Due to missing values (listwise deletion), the size of the sample is lower in some analyzes than stated in this section.

2 The pictorial representations of the animals highlighted the features of each strategy (split strategy as monkey, stepwise strategy as mouse, compensation strategy as squirrel, indirect addition as frog and standard written algorithm as owl). For instance, the indirect addition was labeled as the frog strategy since it just needs a small “jump” from the subtrahend to the minuend to solve suitable subtraction tasks.

3 The within-comparisons in the blocked condition and the between-comparisons in the interleaved condition were carried out using several subtraction tasks in each case.

4 All subtraction tasks were three-digit except of two two-digit tasks in the pretest.
