# Corrigendum: Interleaved Learning in Elementary School Mathematics: Effects on the Flexible and Adaptive Use of Subtraction Strategies

**DOI:** 10.3389/fpsyg.2019.02296

**Published:** 2019-10-18

**Authors:** Lea Nemeth, Katharina Werker, Julia Arend, Sebastian Vogel, Frank Lipowsky

**Affiliations:** Department of Empirical Educational Research, Institute of Educational Science, Faculty of Human Sciences, University of Kassel, Kassel, Germany

**Keywords:** interleaved practice, comparison, subtraction strategies, mathematics, elementary school, strategy-specific adaptivity, flexibility

In the original article, there was an error. We cited the results of Brunmair and Richter's meta-analysis presented on conferences, which did not show an effect of interleaving mathematical tasks over all included studies. Recently, the meta-analysis was accepted for publication in Psychological Bulletin. In this version of the article, more studies were included in the meta-analysis and a small, significant effect of interleaving mathematical tasks was found. Thus, we want to correct our report on this meta-analysis.

A correction has been made to ***Introduction***, ***Interleaved practice and the role of comparisons***, ***Paragraph 2***:

Empirical findings regarding the effectivity of interleaved practice in mathematics are inconsistent, and this is emphasized by Brunmair and Richter's (2019) meta-analysis. This meta-analysis showed a small positive effect of interleaving mathematical tasks on students' procedural knowledge. However, the results of the studies included in this meta-analysis vary strongly. While some found a positive effect of interleaved practice (Rohrer and Taylor, 2007; Taylor and Rohrer, 2010; Sana et al., 2017), others showed no effect or even a negative impact (Rau et al., 2010; Higgins and Ross, 2011). Hence, it can be assumed that the effectivity of interleaved practice in mathematics depends on the concrete design (e.g., implementation, characteristics of learning materials, similarity of categories).

Related to the same issue, a correction has been made to ***Discussion***, ***Paragraph 4***:

“Summarizing the results, interleaving subtraction strategies with supporting discrimination processes by prompts to compare seems to foster the flexible strategy use and the ability to choose an appropriate strategy based on specific tasks and their characteristics sustainably. Therefore, this study supplements previous research on interleaved practice in mathematics, which did not thoroughly show positive effects (Brunmair and Richter, 2019).”

In addition, there was another error. We stated that the stepwise strategy was not part of the analysis to answer research questions 3 and 4. However, the split strategy was excluded from the analysis, while the stepwise strategy was included.

A correction has been made to ***Materials and Methods***, ***Analysis***, ***Research Questions 3 and 4***, ***Paragraph 1***:

To address the third research question, a hierarchical cluster analysis (Ward's method with squared Euclidean distances) was conducted to find out whether there are specific subgroups of students that differ in using the standard written algorithm, the stepwise strategy, the compensation strategy, and the indirect addition adaptively at the points of measurement. The split strategy was again not part of the analysis since it could not have been used adaptively in the strategy test.

The authors apologize for these errors and state that this does not change the scientific conclusions of the article in any way. The original article has been updated.

References

The following reference has been inserted:

Brunmair, M., and Richter, T. (2019). Similarity matters: a meta-analysis of interleaved learning and its moderators. *Psychol. Bull*. https://doi.org/10.1037/bul0000209. [Epub ahead of print].

The following references have been removed:

Brunmair, M., and Richter, T. (2017). Verschachtelung nicht ohne wenn und aber: Eine Mehrebenen-Metaanalyse zu Moderatoren des verschachtelten Lernens [interleaving not without fuss or quibble. a multilevel-meta-analysis on moderators of interleaved practice, abstract]. *Paper Presented at the Gemeinsame Tagung der Fachgruppen Entwicklungspsychologie und Pädagogische Psychologie*, Leipzig.

Brunmair, M., and Richter, T. (2018). Generalisierbarkeit und Wirkmechanismen des verschachtelten Lernens: Eine metaanalytische Untersuchung [generalizability and mechanisms of action of interleaved practice: a meta-analytic investigation, abstract]. *Paper Presented at the 6th Tagung der Gesellschaft für Empirische Bildungsforschung*, Heidelberg.
